# Rubber, or Coffer Dam, Barnums'

**Published:** 1867-05

**Authors:** 


*Rubber, or Coffer Dam, Barnums.'*—From the experience we have had in the use of this simple appliance, as a protection against saliva during the operation of filling certain cavities in the teeth, the approximal for example, we feel disposed to recommend its use to those who have not yet given it a trial. This appliance consists of nothing more than a thin sheet of India-rubber of good quality, that it may not tear easily, and of a thickness double that of letter paper.

The sheet of rubber is cut from four to eight inches square, and near its centre one, two or more holes are made, through which one or more of the crowns of the teeth are passed, when it is applied to the mouth. The holes should be much smaller in diameter than the necks of the teeth they are to embrace; it has been recommended to make them one tenth smaller, and to have several holes in the sheet in order that one or more teeth on either side of the one to be filled, may be included; also, when the teeth approximate closely, to have the holes one eighth of an inch apart, and a greater distance if there is more space between the teeth. The holes should be cut perfectly round, in order that their margins may embrace the necks of the teeth closely, and also to prevent their tearing when dilated. To cut the holes perfectly, some stretch the rubber over a pointed piece of wood which is then cut off with scissors: but a better plan for securing a perfectly round, clean cut hole is to use a small punch with a chisel edge. To carry the rubber thus prepared, between the teeth, waxed-floss silk, or a thin flat burnisher may be used, and the margin of the hole pressed gently under the free edge of the gum in the direction of the root of the tooth. Where care is taken in removing the rubber from the teeth, the same sheet will answer for many operations. It is also economical to make the holes in the rubber sheet near to one of the sides, instead of near the centre, and should rupture of the margins occur, this part of the sheet can be cut away, and the remaining portion used again. As the odor of the rubber may be unpleasant, especially when it is applied to the front teeth, this effect may be obviated by the use of a perfume bag around which the sheet may be wrapped when not in use.

